# Biochar and biosorbents derived from biomass for arsenic remediation

**DOI:** 10.1016/j.heliyon.2024.e36288

**Published:** 2024-08-20

**Authors:** Gaurav Sharma, Yaksha Verma, Chin Wei Lai, Mu. Naushad, Jibran Iqbal, Amit Kumar, Pooja Dhiman

**Affiliations:** a International Research Centre of Nanotechnology for Himalayan Sustainability (IRCNHS), Shoolini University of Biotechnology and Management Sciences, Solan, 173229, Himachal Pradesh, India; b Nanotechnology & Catalysis Research Centre (NANOCAT), Institute for Advanced Studies (IAS), University of Malaya (UM), 50603, Kuala Lumpur, Malaysia; c Department of Chemistry, College of Science, King Saud University, P.O. Box 2455, Riyadh, 11451, Saudi Arabia; d Department of Environmental Sciences and Sustainability, College of Natural and Health Sciences, Zayed University, Abu Dhabi, 144534, United Arab Emirates

**Keywords:** Adsorbents, Adsorption, Arsenic, Biomass, Wastewater

## Abstract

Global groundwater contamination by Arsenic (As) presents a grave danger to the health of living beings and wildlife, demanding comprehensive remediation strategies. This review delves into the complex landscape of arsenic remediation, encompassing its chemical forms, occurrences, sources, and associated health risks. Advanced techniques, notably biomass-derived adsorbents, emerge as promising and cost-effective solutions. The exploration spans preparing and modifying biomass-derived adsorbents, unraveling their adsorption capacity, influencing factors, isotherms, kinetics, and thermodynamics. Noteworthy attention is given to plant-agricultural waste, algal-fungal-bacterial, and iron-modified biomass-derived adsorbents. The comprehensive discussion of the adsorption mechanism highlights the efficacy of low-cost biomass, particularly from plant, animal, and agricultural residues, offering a sustainable remedy for arsenic removal. This insightful review contributes to the understanding of evolving technologies essential for addressing arsenic contamination in wastewater, emphasizing the potential of renewable biomaterials in advancing efficient remediation practices.

## Highlights

• Global arsenic contamination requires cost-effective solutions.
• Biomass combats toxic arsenic contamination effectively.
• The review explores biomass remedies; and highlights arsenic adsorption.
• pH, and isotherms guide efficient biomass adsorption.

## Graphical abstract

**Figure:**
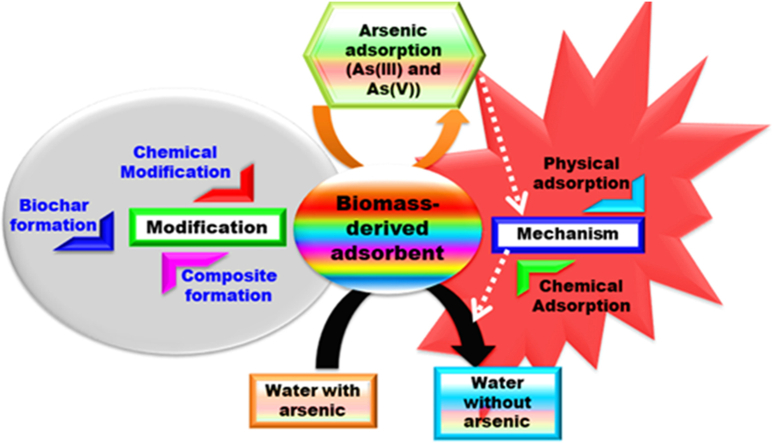

## 1 Introduction

The global freshwater challenge spurred by population growth, urbanization, industrialization, deforestation, and more, demands immediate attention. Effective solutions require prompt and scientifically informed actions. The situation is exacerbated by rising reliance on groundwater and variables such as population development, global warming, and deteriorating water quality. According to WHO and UNICEF, 700 million people do not have access to safe drinking water, and forecasts indicate that 3 billion people may confront water scarcity by 2025 [1]. The impending threat of heavy metal contamination parallels the desire for clean water. Arsenic, cadmium, fluoride, nickel, and mercury pose health concerns even in trace amounts over time. Due to their toxicity and environmental persistence, heavy metals surpass safe levels, producing major health risks [2,3].

Arsenic a metalloid with atomic number 33, is a member of the Nitrogen family and is the 12th, 14th, and 20th most prevalent element in the human body, saltwater, and Earth’s crust, respectively. It can exist in four oxidation states: -3 (arsine), 0 (elemental arsenic), +3 (arsenite), and +5 (arsenate) [4]. In aqueous systems, inorganic arsenic exists in two forms: trivalent arsenic (III) and pentavalent arsenic (V). In aerobic environments, pentavalent arsenic exists stably in its monovalent form or divalent anions, but in an oxygenic situation, trivalent arsenic remains stable in its neutral form or anionic species. Arsenic, with an atomic weight of 74.922 g/mol and a specific gravity of 5.73 g/cm^3^, is a metalloid renowned for its hardness and brittleness [5]. Arsenic, a very hazardous and carcinogenic element, accounts for around 0.00005 % of the earth’s surface [6]. Forest fires, weathering, erosion, and volcanic eruptions are some of the natural processes that release arsenic into the environment. Furthermore, industrial processes like glass processing, alloying, and application of pesticides add to the pollution caused by arsenic [7].

Significant health hazards associated with arsenic-contaminated water include kidney, bladder, skin, and cardiovascular illness as well as impacts on reproduction, diabetes, and abdominal pain. Arsenic is classified as a Group A carcinogen [8] and the World Health Organization (WHO) and the United States has established a safe threshold of 10 μg/L for arsenic in drinking water [9]. Notably, higher quantities of arsenic are permitted in some nations, such as Bangladesh, China, and others, which contributes to health hazards [10]. The World Health Organization’s December 2022 report emphasizes increased exposure to inorganic arsenic, stemming from sources like contaminated water, food preparation, industrial processes, and tobacco use [7]. The urgency lies in developing sustainable methods for arsenic decontamination, applicable at household and community levels [7]. Different ways to remove arsenic from water include ion-exchange [11], coagulation [12], flocculation [13], oxidation [14], adsorption [15], precipitation [16], and membrane procedures [17,18].

Adsorption is a potential technology for arsenic remediation and harmful pollutant elimination [[19], [20], [21]]. It makes use of various adsorbents such as activated charcoal [22], zeolites [23], Silica- Iron Oxide Nanocomposite [24], activated cellulose fibers [25] and carbon nanotubes [26], etc. Adsorption removes arsenic from wastewater with benefits such as sludge-free operation and cost-effectiveness. This versatile approach is suitable for both massive and small-scale applications, which is especially advantageous for developing countries. While typical adsorbents suffer from challenges such as high prices and inadequate removal, adsorption using biomass-based adsorbent emerges as a viable solution for containments, notably arsenic.

Biomass, which includes agricultural byproducts, urban refuse, industrial effluents, and forest remnants, is a low-cost, ecologically beneficial adsorbent (biosorption) for water treatment [[27], [28], [29], [30]]. Because of its porous structure, short regeneration period, efficacy, and various functional groups, it can adsorb contaminants from wastewater. Biomass-based adsorbents are garnering interest and are being extensively researched for the removal of arsenic due to their favorable surface properties, affordability, and environmental friendliness.

This extensive analysis’s focal point revolves around eliminating arsenic utilizing adsorbents derived from biomass. Our focus extends to recent advancements in biomass-based adsorbents, exploring adsorption mechanisms, factors influencing arsenic removal in adsorption systems, and the sources of arsenic pollution. Finally, we present future perspectives in this critical area of research.

### 1.1 Chemical forms, occurrence and distribution of arsenic in water

**Fig. 1:**
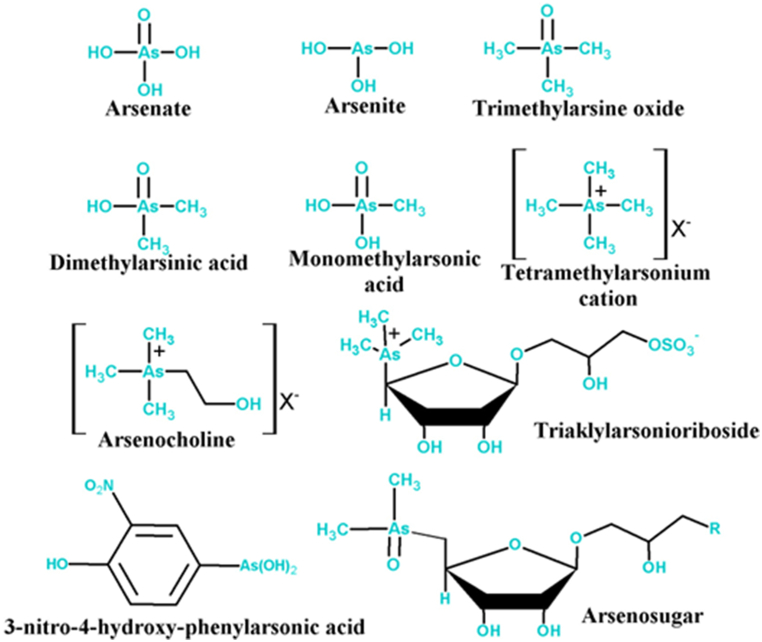
Various As species found in nature.

Arsenic is found in soil, sediments, and water all over the global community, comes from both naturally occurring and anthropogenic sources, and exists in four different oxidation levels (0, −3, +3, and +5). Arsenite (As (III), H_3_AsO_3_^−^) and arsenate (As (V), HAsO_4_^2−^) are chiefly common elements found in nature [31]. Arsenic (V) predominates in oxidizing settings, creating arsenic acid oxyanions (H_3_AsO_4_, H_2_AsO_4_^−,^ HAsO_4_^2−^, AsO_4_^3−^), while arsenic (III) is prevalent in moderately reducing environments as arsenious acid (H_3_AsO_3_, H_2_AsO_3_^−^, HAsO_3_^2−^) and is thermodynamically more stable [32]. Natural water bodies also harbor organic arsenic compounds, including DMA-III (dimethylarseneous acid (III)), MMA-III (monomethyl arseneous acid (III)), MMA-V (monomethyl arsenic acid (V)), and DMA- V (dimethyl arsenic acid (V)) [33]. Fig. 1 represents various arsenic species found in nature. These organic arsenic forms are typically found in concentrations lower than 1 μg per liter and generally do not hold significant importance within the framework of drinking water purification. In prior investigations exploring the influence of pH on the speciation of arsenic, distinct chemical kinds of arsenic exhibit varying prevalence within specific pH ranges. The presence of arsenic in groundwater is affected by numerous factors such as its sources, redox conditions, groundwater flushing, bioavailability of organic matter, and the distribution of clay/peat layers [34,35]. Higher concentrations of arsenic in groundwater are often reported under reducing conditions. Within the pH spectrum of 5.0–8.0, the predominant types of As(V) are H_2_AsO_4_^−^ and HAsO_4_^2−^, while As(III) is primarily present as H_3_AsO_3_ at pH ≤ 9.2 [36]. Consequently, negatively charged arsenic (V) is prone to adsorption onto sediments more readily than arsenic (III) due to its neutral charge. When subjected to oxidative conditions, HAsO_4_^2−^ becomes the dominant form, particularly in high pH environments. Notably, extremely acidic and alkaline conditions contribute to higher concentrations of H_3_AsO_4_ and AsO_4_^2−^. In instances of low pH (<6.9), H_2_AsO_4_ prevails as the dominant species. This underscores that, under such conditions, arsenic (III) persists as an uncharged molecule in natural aquatic systems [37]. It is widely acknowledged that the reduced form, arsenic (III), is more harmful, soluble, and mobile compared to the oxidized form, arsenic (V). Organic arsenic compounds are typically less harmful than their inorganic counterparts. In investigations exploring pH impact on arsenic speciation, distinct chemical forms of arsenic prevail within specific pH ranges, emphasizing the importance of environmental conditions in understanding arsenic behavior in water systems.

*Arsenic in groundwater:* Internationally, many countries adhere to a standard limiting, setting a maximum threshold of 10 μg/L for arsenic in groundwater. Arsenic pollution in groundwater at high quantities has been widely reported in several countries, including nations, encompassing the United States, Argentina, China, Bangladesh, Chile, Mexico, and India, with values spanning from 1 μg/L to 73.6 mg/L [31,38]. However, in regions facing more pronounced arsenic pollution, as observed in Bangladesh and various other Asian countries, the permissible maximum level of arsenic in drinking water is set at 50 μg/L. This reflects the recognition of varying environmental conditions and the need for tailored regulations to address heightened arsenic concerns in specific areas.

Arsenic *in surface water:* The typical baseline concentration of arsenic in rivers falls within the range of 0.1–2.0 μg/L [39]. Regulations for arsenic levels in lakes usually aim for concentrations less than 1 μg/L, with some tolerance for levels close to 1–2 μg/L [39]. However, there have been instances of elevated arsenic concentrations in lakes globally. For example, Taihu Lake (China) exhibited arsenic content ranging from 1.39 to 6.65 g/L, while Dianchi Lake (China) showed concentrations from 3.08 to 10.48 g/L [40]. Due to geothermal and human influences, arsenic concentrations in the Gomati river (Ganga Plain, northern India) ranged from 1.29 to 9.62 μg/L [41].

Arsenic *in seawater:* The concentration of arsenic in seawater is typically below 2 μg/L [42], as indicated in various studies. Along the deep Pacific and Atlantic coasts, the concentration of arsenic falls within the range of 1.0–1.8 μg/L. Similarly, off the coast of Malaysia, arsenic concentrations range from 0.7 to 1.8 μg/L, and off the coast of Spain, they vary between 0.5 and 3.7 μg/L [43]. These measurements underscore the generally low levels of arsenic in seawater across different geographical locations, contributing to our understanding of the baseline concentrations of this element in marine environments.

### 1.2 Sources of arsenic contaminants

**Fig. 2:**
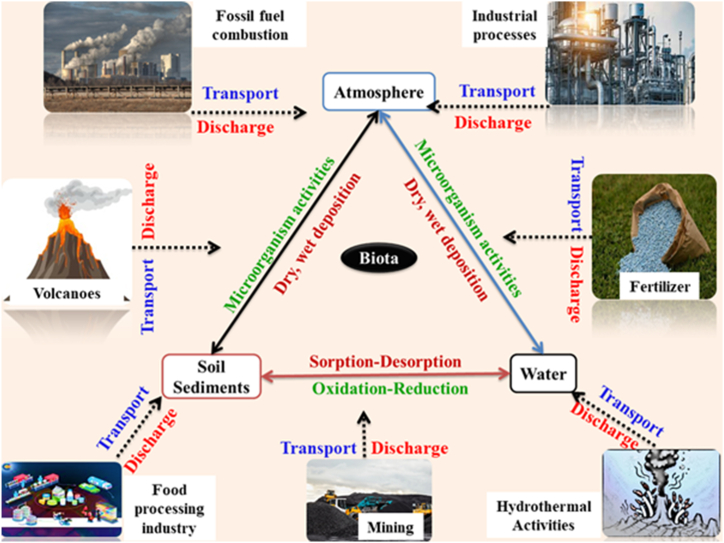
Sources of arsenic and arsenic cycle.

Arsenic pollution is a global hazard, produced by a combination of man-made and natural factors. Geological processes like weathering, abrasion, volcanic eruptions, hydrothermal processes, and sediment deposition are examples of natural sources. The processing of minerals and other anthropogenic activities like mining greatly increases the risk of arsenic contamination. Certain rocks and minerals, such as AsS (realgar), pyrite, As_2_S_3_ (orpiment) and FeAsS (arsenopyrite), can leak arsenic into the surface or groundwater. Natural waterways have varying concentrations of arsenic due to hydrogeological conditions, local geology, pH, and redox potential [36,[44], [45], [46], [47]]. Fig. 2 given below represents sources of arsenic and arsenic cycle.

*Human-induced* arsenic *release:* Mining, industrial operations, intensive farming, use of fertilizers and pesticides, and other human activities, cause arsenic to be released into groundwater. The quality of aquifers is impacted by the discharge of arsenic caused by the burning of fossilized fuels and the utilization of wood preservatives [45]. When fossil fuels are consumed, anthropogenic interventions cause As_4_O_6_ to volatilize and condense into groundwater storage [48]. To ensure the safety of the water supply and to implement appropriate mitigation techniques, it is essential to comprehend both natural and manmade sources.

*Geological formations and* arsenic: More than 210 minerals contain arsenic, most of which are sulfides like pyrite and arsenopyrite. Groundwater levels of arsenic are elevated due to desorption and dissolution of these minerals, particularly in deltas and alluvial plains [46,48,49]. Sediments with high concentrations of iron oxide or hydrous metal oxide exhibit elevated levels of arsenic. Arsenic release and mobility are influenced by a major mechanism of reduction of iron and aluminum metal oxides and the activity of native metal-reducing bacteria [50].

*Oxidation States and Speciation:* Arsenic is mainly found in groundwater as oxy anions, corresponding to the oxidation states arsenic (III) and arsenic (V). The pH spectrum from 6 to 9 has an impact on the prevailing form [48]. Arsenic (III) is considered more dangerous and challenging to eliminate compared to arsenic (V). The dissolution/desorption processes and the arsenic content of the aquifer are blamed for the fluctuations in arsenic concentration. One of the main reasons why arsenic leaks out of aquifer sediments is a reductive breakdown of iron oxide [51].

An in-depth analysis of the geological, anthropogenic, and speciation components of the various sources of arsenic pollution is provided in this paper, which is crucial for developing mitigation and management plans that work.

### 1.3 Health risks

**Fig. 3:**
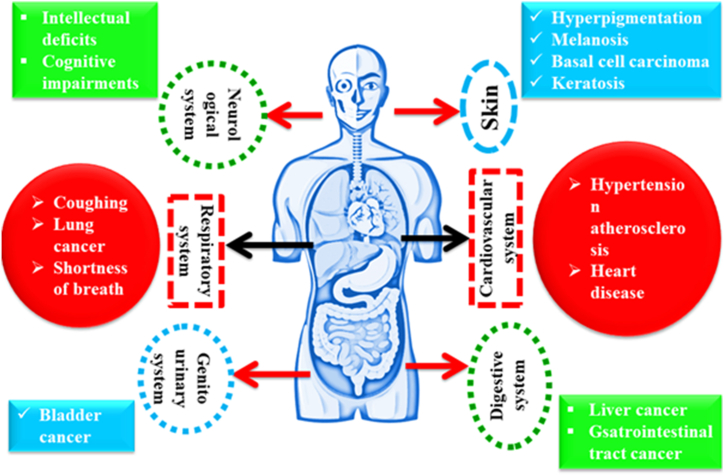
Health issues caused by arsenic.

Arsenic infiltrates the human body via two primary routes: consuming contaminated water and eating arsenic-rich foods. More than 200 million people worldwide face health concerns as a result of arsenic contaminated water intake. WHO advises that continuous exposure to arsenic levels exceeding 50 μg/L can result in skin and visceral malignancies, diabetes, hypertension, and reproductive system abnormalities. The primary cause of arsenic exposure is drinking water, which damages the central nervous system (CNS), kidneys, dermis, liver, and pulmonary system. Chronic arsenic toxicity also has an impact on cardiovascular health and can cause skin discoloration and hard patches on the palms after extended exposure to contaminated water [[52], [53], [54]]. Fig. 3 given below shows various health issues caused by arsenic.

## 2 Technique for arsenic remediation

**Fig. 4:**
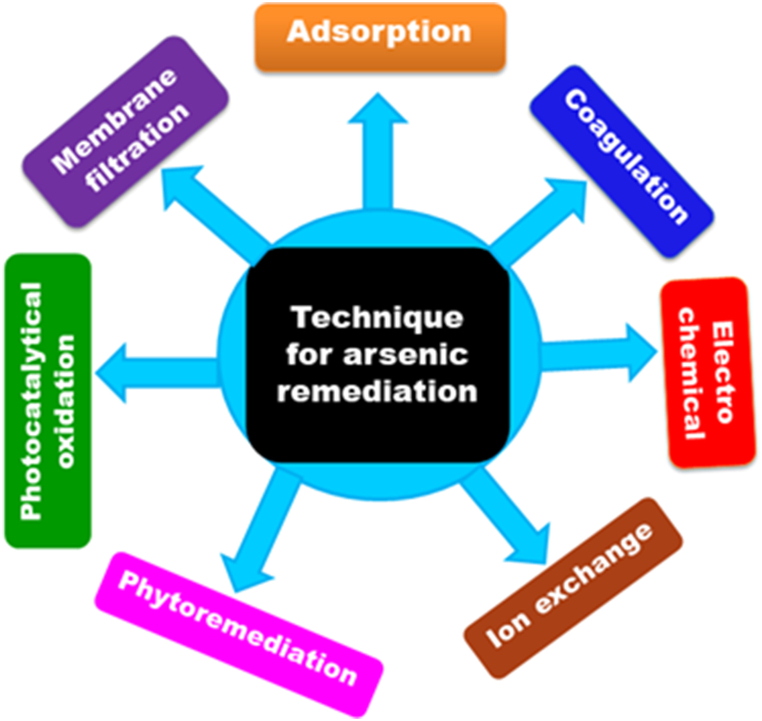
Technique for arsenic remediation.

Given the dangers of arsenic in water, particularly drinking water, numerous techniques for its removal have been investigated. For successful arsenic removal, conventional methods including membrane processes, electrochemical techniques, coagulation, ion-exchange, phytoremediation, and adsorption (Fig. 4) have been extensively researched and used.

*Membrane filtration:* Membrane filtration, a popular arsenic removal technology, employs films with selected pores for targeted removal [55]. Ultrafiltration deals with colloids and viruses, nanofiltration with charge and molecular size, microfiltration with bacteria, and hyperfiltration with desalination [56]. The pressure difference-driven method decreases arsenic concentrations to less than 50 μg/L, although at a significant cost and with residual volumes. Nanofiltration and reverse osmosis stand out for their efficiency, ease of use, and low sludge. Several researchers coupled pre-oxidation followed by reverse osmosis membrane for arsenic removal [57]. Initial expenses, high-pressure requirements, fouling of the membranes, and a decrease in flux are all obstacles. Another method is electrodialysis, which eliminates arsenic but has drawbacks such as cathode coagulant deposition [5,58].

*Coagulation:* Coagulation, as proved in several experiments, successfully eliminates arsenic from water by introducing a coagulant [59], often ferric chloride (FeCl_3_). This technique, which targets arsenic in its oxidized state [As (V)], also targets fluoride, manganese, iron, phosphate, and turbidity. In water, FeCl_3_ hydrolyzes to create positively charged ferric hydroxide [Fe (OH)_3_], which attracts and binds negatively charged arsenate [As (V)] particles. To ensure efficiency, any arsenite [As (III)] present may require pre-treatment with chlorine for oxidation. Following sedimentation and filtration operations, the produced arsenic particles are removed from the water.

*Electrochemical techniques:* Electricity was first used in wastewater treatment in the United Kingdom in 1887 [60]. For arsenic removal, various electrochemical techniques such as electrodeposition [61], electrodialysis [62], and electrooxidation [63] have been used. Among these is electrocoagulation, which uses sacrificial electrodes to trigger chemical and physical interactions that result in coagulant production. Even though it requires a significant amount of electricity, electrocoagulation is commonly employed for effective arsenic elimination [64].

*Ion-exchange:* Ion exchange with anion exchange resins is useful for removing arsenic [65], particularly arsenic (V). However, it is less efficient for uncharged arsenic (III) and can be costly. Adsorption restrictions exist due to competition with other anions, and sludge disposal issues arise during regeneration. This physicochemical process includes exchanging ions in a solution with those on a solid resin. It is widely utilized for the treatment of drinking water to soften water and eliminate pollutants including arsenate, nitrate, selenate, and chromate. Strong base anion exchange resins, which are effective throughout pH ranges are commonly used for arsenic removal, although exhausted resin requires extra treatment before disposal or reuse [5,60]. Selective removal of arsenic(V) from drinking water was performed using strong base anion-exchange resins Purolite A-505 and Relite-A-490 [66]. The efficiency depended on the type of resin and water composition. Regeneration with sodium chloride solution allowed arsenic to be precipitated using FeCl_3._ Adding FeCl_3_ in 10–15 times excess and adjusting the pH to 7–8 precipitated over 99 % of the arsenic. The filtered regenerate solution could then be reused after adjusting the sodium chloride concentration. A glycidyl methacrylate/methylene bisacrylamide resin with immobilized tetraethylenepentamine ligand was transformed into two anion exchange resins: RI and RII [67]. These resins adsorbed arsenic(V) effectively, with maximum capacities of 1.83 mmol/g and 1.12 mmol/g, respectively. Regeneration using 1 M HCl showed 99 % efficiency for RI and 98 % for RII, indicating high durability for reuse.

*Phytoremediation:* Phytoremediation, an environmentally beneficial technology, employs vegetation and microorganisms to remediate arsenic-contaminated air, soil, and water with minimal nutrient supplementation [68]. This technology, which is divided into phytoextraction, phytostabilization, phytofiltration, and phytovolatilization, is rooted in plants possessing robust root systems, high resilience, and rapid growth, and arsenic adsorption capacities for long-term environmental remediation [69,70].

*Photocatalytical oxidation:* Surface reactions occur in a photocatalytic system when the photocatalyst captures photons with energy same to or exceeding its bandgap. This process produces electron-hole pairs, with excited electrons going to the conduction band and forming holes with a positive charge in the valence band [71,72]. The electron-hole pairs produced serve a critical part in the fundamental mechanism of photocatalytic responses, promoting the transformation of target pollutants [[73], [74], [75]]. To eliminate arsenic (III) by photocatalytic oxidation, a bi-functional ZrO_2_–Fe_3_O_4_ magnetic nanocomposite was produced and investigated. ZrO_2_–Fe_3_O_4_ nanoparticles could oxidize arsenic (III) under UV radiation in 40 min, resulting in less toxic arsenic (V) [73]. Photocatalytic oxidation is used to convert arsenic (III) to arsenic (V), but this process needs to be followed by a technique specifically designed to remove arsenic (V).

**Adsorption:** Adsorption is a technique that uses solids as a medium to expel compounds. The van der Waals interaction and electrostatic attraction among adsorbent molecules and surface atoms are the primary driving forces behind the entire process. Solids, including synthetic materials (fibers, resins, gels, activated carbon, nanotubes) and natural materials (Biomass), are commonly used in industrial wastewater treatment, but the production of synthetic materials is expensive, energy-intensive, and less environmentally friendly compared to natural materials [76].

The adsorption process is considered highly promising for treating wastewater because of its affordability, high efficiency, and ease of use. As previously stated, numerous techniques have proven useful in eliminating arsenic from water. Adsorption is a well-reviewed treatment technology for removing arsenic(V). Numerous relevant articles have extensively covered its effectiveness and applications [[19], [20], [21]]. This review examines the efficiency of arsenic removal from contaminated drinking water and groundwater using the adsorption technique with biomass-derived materials (biosorption).

## 3 Biomass-derived adsorbent for arsenic remediation

Biomass adsorbents, encompassing materials like corn stalks, animal wastes, fruit peel-off, nut shells, charcoal, peat, chitin, and various wastes, offer versatile solutions for efficient metal ion and contaminants removal across industries. Both live and deceased biomass, including plant-based, algal, fungal, and bacterial biomass, have proven effective in adsorbing arsenic ions, showcasing their significance in environmental and industrial contexts. The inclusion of functional groups such as carboxyl, hydroxyl, and sulfhydryl improves arsenic removal on the surface of the adsorbent, making biomass a non-toxic and robust option for environmental restoration. The adsorption performance of biomass as an eco-friendly and cost-effective adsorbent is determined by structural parameters such as pore distribution and surface area. However, recent studies have highlighted the paramount importance of surface chemistry and surface charge over traditional textural properties like pore distribution and surface area. Specifically, the pHpzc (point of zero charge) is a key parameter for surface characterization of adsorbents, indicating the pH at which the surface is neutral. For example, a shift in pHpzc to lower values after As(III) uptake, as observed in the materials, indicates ion exchange and the formation of inner sphere complexes. This shift confirms the adsorbent’s interaction with As (III) through ion exchange mechanisms. This effect, particularly pronounced in materials like MNP-PCP (magnetite immobilized on pine cone) [77], underscores the significance of pHpzc in understanding the adsorption mechanism and optimizing the material’s performance for arsenic removal.

Additionally, for arsenic (V) removal from aqueous solutions, the surface charge of the adsorbent plays a crucial role. Arsenic (V) exists mainly as negatively charged species (H_2_AsO_4_^−^ and HAsO_4_^2−^) at neutral pH levels, leading to electrostatic interactions with the adsorbent surface. Materials with a higher point of zero charge (pH_PZC_) create a positively charged environment that attracts arsenic anions, enhancing the adsorption capacity. Conversely, negatively charged surfaces repel these anions, reducing the adsorption efficiency.

The presence of iron, especially in the form of iron oxyhydroxide nanoparticles, significantly enhances arsenic adsorption through ligand interchange mechanisms [78]. These iron nanoparticles can replace hydroxyl ligands in arsenate molecules, forming stable complexes on the adsorbent surface. However, for these interactions to be effective, the initial attraction between the arsenic species and the adsorbent is necessary, which is largely governed by the surface charge. Empirical studies have shown that basic activated carbons with an iron content of about 1 % exhibit optimal performance for arsenic removal, combining electrostatic attraction and specific chemical interactions [79]. Therefore, while structural properties like surface area and micropore volume are important, the surface charge, pHpzc and chemical composition of the adsorbent are more critical in determining its effectiveness for arsenic (V) uptake. This underscores the need to consider both surface chemistry and textural properties in designing and optimizing adsorbents for environmental applications.

Temperature, pH, and other factors influence biomass composition. To further understand pollutant binding mechanisms, adsorption procedures must be optimized and thoroughly characterized utilizing techniques such as FTIR, XPS, SEM, XRD, EDX, and surface area analysis.

### 3.1 Preparation and chemical modification of biomass-derived adsorbent

**Fig. 5:**
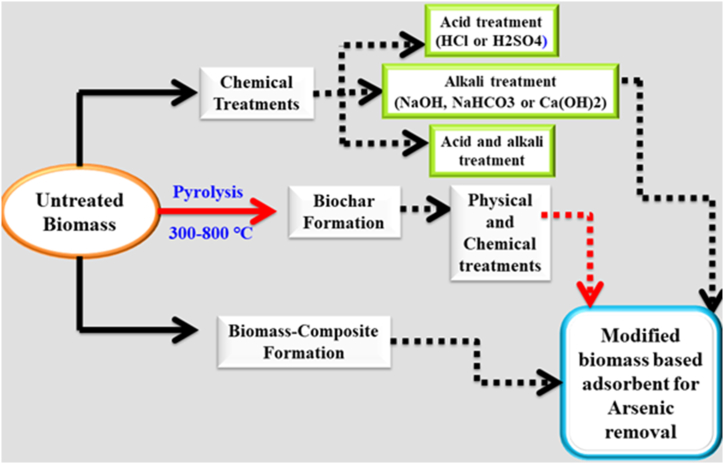
Preparation and chemical modification of biomass-derived adsorbent for arsenic removal.

Optimized biomass-based adsorbent offers advantages like excellent efficacy for minimal metal concentrations and metal recovery, but the unprocessed biomasses require modification to overcome low sorption capacity, stability issues, and environmental concerns before commercial use. Treatment methods for biomass-derived adsorbents include chemical treatment, biochar formation, and biomass composite formation (Fig. 5).

*Chemical treatments* are three types: Acid treatment, alkali treatment, and both acid and alkali treatment. Exposure to solutions with basic or acidic pH during this process undergoes oxidation of the biomass outer layer, forming functional groups containing oxygen, and influencing various physical and chemical characteristics like hydrophilicity, hydrophobicity, elasticity, ion exchange, and sorption capacity [80]. NaOH, NaHCO_3_, and Ca(OH)_2_ are common alkaline agents used for biomass treatment to improve As sorption. Acidic solutions like HCl and H_2_SO_4_ have also been employed to modify biomass to boost arsenic content biosorption. The sequential use of acid and alkali solutions in biomass treatment can create an increased surface area and incorporation of diverse surface-bound functional moieties, potentially increasing the overall sorption capacity of the biomass. Treatment with strong dehydrating agents like H_2_SO_4_ enhances arsenic sorption efficiency through surface area alteration of the sorbent. [81], as demonstrated by Abid et al. Additionally, Yang et al. utilized Ca(OH)_2_ as a saponification agent to activate green-tea biomass for arsenic (III) biosorption under varying pH conditions [82]. Biomass derived from the Neem tree was activated through immersion in NaOH solution and subsequent treatment with H_2_SO_4_, demonstrating ion exchange as a primary mechanism for arsenic (III) removal, as reported by another study [83].

*Biochar formation:* Biochar, a carbonaceous product formed through pyrolysis of different biomass materials under low oxygen conditions, has emerged as a widely used adsorbent due to its significant surface area. It is prepared via slow pyrolysis within a temperature range of 300–800 °C over an extended duration. To further enhance the properties of biochar various chemical and physical treatments have been performed [84,85].

*Biomass composite formation:* Biomass composites are formed by impregnating organic chemicals and metal oxides into biomass, altering its structure and increasing sorption capacity for improved adsorption performance. For arsenic removal, mostly iron-impregnated biomass composites are the most popular or most common compared to other biomass composites. Wu et al. investigated arsenic (V) biosorption in eucalyptus leaves coated with iron nanoparticles. As the sorption mechanism, they observed the coordination of ferric iron nanoparticles with arsenic (V) and the formation of a bidentate binuclear complex and monodentate chelating ligand [86]. In another study [77,87], Pine cone biomass underwent activation through immersion in a sodium nitrate solution. The treatment involved the deposition of NO_3_^−^ onto the surface of the biomass, which was subsequently replaced by arsenic (III) during the sorption process via an ion exchange mechanism. This process led to an enhancement in the sorbent’s capacity for arsenic (III) sorption. There are various examples of biomass composites for arsenic removal in the literature [[88], [89], [90]].

### 3.2 Adsorption capacity, factors affecting adsorption, isotherms, kinetics, and thermodynamics

#### 3.2.1 Adsorption capacity (q_t_)

It is a critical aspect, to study the adsorption of arsenic onto the sorbent material. It is explained as the quantity of adsorbate (arsenic) adsorbed per unit mass of adsorbent (biomass-derived adsorbent) [91]. Experimental adsorption capacity is determined by following the equation given below,

$$q_{t}=\frac{\left(\;C_{o}-\;C_{t}\right)V}{m}$$

where, $q_{t}$ is the amount of arsenic adsorbed on biomass-derived adsorbent at time t; $C_{o}$ (mg/liter) is the starting amount of arsenic and $C_{t}$ (mg/liter) is the arsenic quantity at time t; m (mg) is the biomass-derived adsorbent mass used, and V (mL) is the solution volume. Adsorption isotherms are also utilized to ascertain the maximum equilibrium capacity of adsorption which we will discuss later in this section.

#### 3.2.2 Factors affecting adsorption of arsenic

The various factors that influence the adsorption of arsenic via biomass-based adsorbent involve pH, adsorbent concentration, adsorbate concentration, and the effect of counter ions.

pH: One of the critical elements in the sorption of arsenic species is aqueous solution chemistry, which has a major influence on biomass surface characteristics, such as the electrochemical charge of biomass under varying pH conditions and arsenic speciation [92]. The interaction of biomass and arsenic types was influenced by the point of zero charges (PZC). When the pH at the point of zero charge (_PZC_pH) exceeds the solution pH, it favors cation adsorption via electrostatic contact, while adsorption of anion is preferred in the solution when pH exceeding _PZC_pH of biomass [93].

*Adsorbent concentration:* Optimizing the adsorbent dose, whether it is biomass or its modified form, is crucial for achieving maximal arsenic remediation, maintaining constant initial arsenic concentration and pH conditions. The remediation efficacy exhibits an ascending trend with escalating biomass doses, reaching an optimum level where saturation occurs indicating that every active sites on biomass outer layer is effectively utilized [94,95].

*Adsorbate concentration:* The capacity of adsorption of biomass-derived sample is intricately tied to the initial arsenic amount in the solution with water, exerting a substantial impact on the procedure dynamics. When the biomass dose remains constant and the starting arsenic amount is minimal, reactive sites on the biomass surfaces remain readily available for arsenic ions. Contrastingly, as the arsenic concentration rises without a corresponding increase in biomass doses, the adsorption efficiency diminishes, attributed to the diminished presence of reactive sites and intensified competition within ions of arsenic [29,96,97].

*Counter ions:* The existence of various typical ions including phosphate, carbonate, sulfate, chloride ions, bicarbonate, and others, especially in groundwater due to anthropogenic and geochemical processes, can adversely affect arsenic elimination [98]. This is due to the competitive character of these ions, which might obstruct arsenic adsorption by competing for accessible binding sites. Adsorption efficiency due to electrostatic interactions, the adsorption of arsenic onto the biomass-derived adsorbent is influenced by the presence of co-existing ions. Specifically, negatively charged ions (NO_3_^−^, SO_4_^2−^, PO_4_^3−^) compete with arsenic anions for binding sites on the adsorbent surface, potentially hindering arsenic uptake. Conversely, positively charged cations (Mg^2+^, Ca^2+^, Mn^2+^) may experience electrostatic repulsion from the positively charged surface, leading to less interference with arsenic adsorption [99]. The phosphate and silicate ions pose a greater challenge for arsenic ions in terms of adsorption carbonate and sulfate due to the similarity in between oxyions of arsenic, phosphate and silicates [4,100]. Carbonate in groundwater can affect the adsorption of arsenic. In natural water, dissolved carbonate primarily exists as HCO₃⁻ (bicarbonate). High concentration of carbonate significantly influence the arsenic (III) adsorption [101]. Bicarbonate (HCO_3_^−^) alone has minimal impact on arsenic (As) adsorption in geogenic arsenic-contaminated groundwaters. However, when HCO_3_^−^ coexists with Ca^2+^ and Mg^2+^, it significantly enhances the release of adsorbed arsenic, more than competitive effects alone. This rapid desorption of arsenic (V) is attributed to the formation of an aqueous HCO_3_–Ca–As(V) complex, confirmed by recent spectroscopic techniques. The combined influence of HCO_3_^−^ and Ca^2+^ on arsenic immobilization in groundwater remains uncertain [102]. One study has shown that HCO_3_^−^ caused less desorption of arsenic as compared to PO_4_^3−^ [103].

#### 3.2.3 Adsorption isotherm, adsorption kinetics, and adsorption thermodynamics

*Adsorption isotherm:* To assess the adsorbent’s effectiveness, an adsorption isotherm was employed. These isotherm models depict the equilibrium relationship between the adsorbed molecule (adsorbate) and the adsorbent surface at a constant temperature [104]. Several well-established adsorption isotherm models, including Langmuir, Freundlich, Temkin, Sips, and Dubinin-Radushkevich (D-R), are commonly employed to analyze the interaction between arsenic (As) and biomass-derived adsorbents. These models provide valuable insights into the adsorption behavior at the arsenic-biomass interface. Out of the entire isotherm model Langmuir (monolayer adsorption), and Freundlich (multilayer adsorption) isotherm models have been widely used [105,106]. A. Sarwar et al. studied the efficacy of arsenic removal from *Melia azedarach* biomass. Under optimal conditions, the investigation found that arsenic (III) and arsenic (V) were removed at rates exceeding 90 %. The adsorption data was fitted to both Langmuir and Freundlich isotherm models to assess their applicability. Based on R^2^ value, it was established that the Freundlich isotherm was most suited for arsenic (III) and arsenic (IV) adsorption, revealing the multilayer nature of the adsorption [107].

*Adsorption kinetics:* It charts the progression of adsorption with time, under controlled conditions of temperature and pressure [108]. Predicting the rate of adsorption is critical for selecting adsorbent size and residence duration in an adsorption system [109,110]. Understanding and predicting the dynamics of adsorption and the factors that govern its rate mechanisms requires kinetic analysis, specifically employing pseudo-1st-order, pseudo-2nd-order, and intraparticle diffusion. Physisorption and chemisorption generally exhibit pseudo-1st-order and pseudo-2nd-order kinetic behavior, respectively. Intraparticle diffusion models (Weber-Morris model) focus on the internal diffusion of adsorbate molecules within the adsorbent particle as the rate-limiting step, incorporating the influence of surface diffusion and mass transfer at the particle boundary. The Weber-Morris model describes intraparticle diffusion in adsorption systems, represented by a linear plot of the amount of adsorbate against the square root of time. It accounts for the kinetic behavior of sorbents, showing that linearity indicates intraparticle diffusion as the rate-limiting step. However, the model’s linearity is influenced by the adsorbent particle size, with smaller particles possibly involving faster kinetic mechanisms. Additionally, the model suggests that surface energetic heterogeneity and the equilibrium adsorption isotherm type do not significantly impact its linearity, making it applicable to various adsorption systems. For systems with finite bulk phase volumes, the linearity range is more restricted compared to idealized systems with constant adsorbate concentrations [111]. Adsorption kinetics of arsenic (V) eliminated via cattle bone char was evaluated using pseudo-1st and pseudo-2nd-order kinetics. Arsenic (V) adsorption process exhibited pseudo-2nd-order reaction kinetics, as evidenced by high R^2^ values exceeding 0.99. So, arsenic (V) adsorption via cattle bone char is considered to exhibit chemisorption.

*Adsorption thermodynamics:* Understanding the contribution of temperature on adsorption behavior is crucial, and thermodynamic parameters contribute substantially to elucidating the spontaneity of these processes. Also, the activation energy for arsenic adsorption indicates the energy barrier that must be overcome for the adsorption process to occur. Low activation energy values (<42 kJ/mol) typically suggest diffusion-controlled processes, while higher values (>42 kJ/mol) indicate chemically controlled processes. Negative activation energy suggests that increasing the temperature does not favor adsorption, indicating an exothermic reaction [112]. These thermodynamic parameters encompass alterations in ΔH (enthalpy-change), ΔS (entropy-change), ΔG (Gibbs free energy-change), and Ea (activation energy), provide valuable insights into the thermodynamic feasibility of adsorption phenomena.

These essential parameters hold significance in our comprehensive exploration of diverse adsorbents derived from biomass for removal of arsenic. We will delve into a discussion on each, unraveling their role in enhancing arsenic removal efficacy.

### 3.3 Plant, animal, and agricultural waste biomass-derived adsorbents

Employing agricultural by-products, animal wastes, and botanical entities for arsenic sequestration offers an innovative, eco-friendly alternative to conventional wastewater treatment. Research explores the efficacy of diverse biomass materials, like seeds, straw, fruit peels, and more, providing a sustainable and economically viable strategy for arsenic removal. This approach leverages readily available resources, aligning with environmental responsibility and cost-effectiveness in combating arsenic contamination in aqueous environments.

**Fig. 6:**
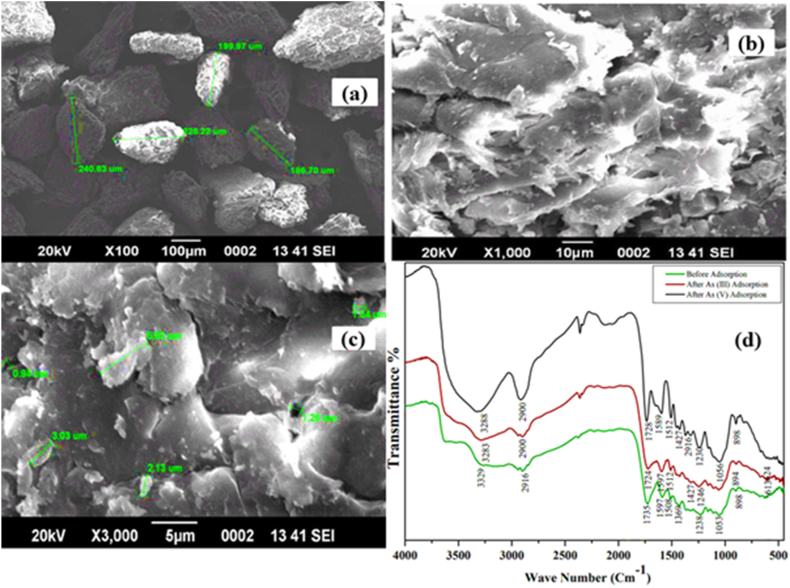
Biochar derived from *Cassia fistula*: (a–c) SEM images; (d) FTIR spectra pre and post-adsorption (arsenite and arsenate). Reprinted with the permission from Ref. [113].

In the pursuit of effective adsorbents derived from plant, animal, and agricultural waste biomass for arsenic species removal, the study investigated the biosorption capabilities of ***Cassia fistula***-based biochar, a member of the *Fabaceae* family. SEM/EDX characterization revealed the activated biochar’s unique non-uniform surface with internal voids, enriched incorporating Ca and Fe to enhance arsenic binding. Fig. 6(a–c) showed the SEM images of ***Cassia fistula*** based biochar. FTIR analysis confirmed surface functionalization with groups that facilitate arsenic interaction and capture (Fig. 6d). Adsorption studies conducted in a batch mode systematically optimized parameters such as contact time (40 min), adsorbent dose (6 g/L (arsenite) and 4 g/L (arsenate)), 27 °C temperature, initial arsenic concentration, pH (6 for As (III) and 2 As (V)), and stirring rate. Under optimal conditions, the activated biochar exhibited notable removal percentages, reaching a maximum adsorption capacity of 0.78 mg/g (78.1 % removal efficiency) for arsenic (III) and 0.42 mg/g (84.8 % removal efficiency) for arsenic (V). The widely used Freundlich-isotherm, indicating a multistep binding process demonstrated excellent fitting, and Dubinin–Radushkevich model fitting suggested physisorption. Kinetics studies supported a pseudo-2nd-order reaction mechanism, while thermodynamic data revealed a negative ΔG adsorption process. Phosphate was identified as the ion most hindering arsenic removal. Regeneration studies showcased promising recovery percentages 23.0 % for arsenic (III) and 21.1 % for arsenic (V). This suggests good resistance to leaching across a range of pH values [113].

**Fig. 7:**
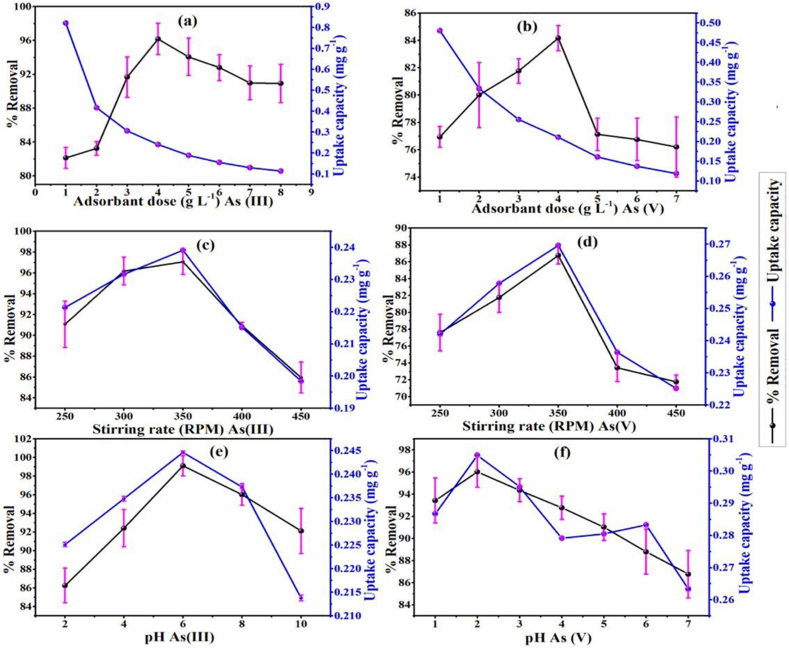
Optimized parameters for As (III) and As (V): (a and b) the adsorbent dosage; (c and d) the stirring rate; (e and f) pH, respectively. Reprinted with the permission from Ref. [99].

**Fig. 8:**
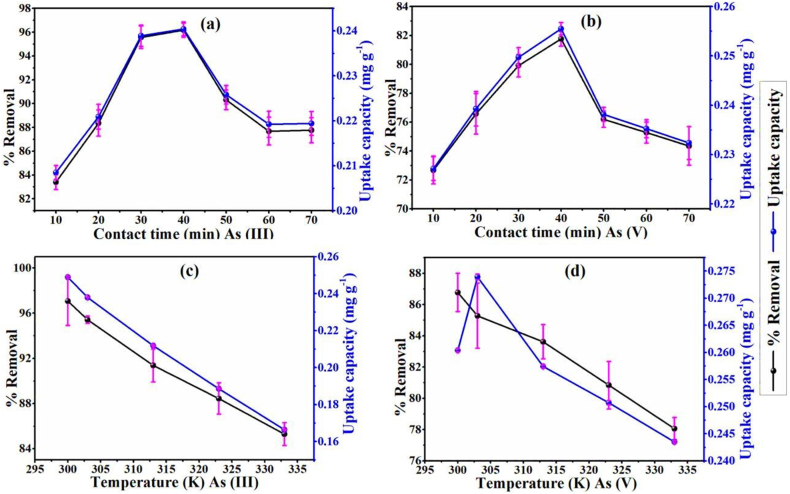
Optimized parameters for As (III) and As (V): (a and b) contact time; (c and d) the temperature, respectively. Reprinted with permission from Ref. [99].

In comparison, Shaikh et al. explored the development and utilization of zero-valent Fe nanoparticles (nZVI) in a biochar-based iron nanocomposite (nZVI/BC) for efficient arsenic removal from aqueous solutions. nZVI/BC, prepared by precipitating nZVI onto biochar derived from ***Cassia fistula*** pods, exhibited remarkable adsorption efficiency for both arsenic ions [99]. The Langmuir model revealed maximum adsorption of 1.04 mg/g (As (III)) and 1.40 mg/g (As (V)). Optimized parameters involve contact time of 40 min, temperature 300 K, pH 2 (As (V)) and 6 (As (III)), homogenization speed 350 rpm, and dosage of the adsorbent material 5 (As (V)) and 4 (As (III))g/L (Fig. 7, Fig. 8d). Optimized conditions resulted in remarkable removal efficiencies, exceeding 99.1 % for arsenic (III) (initial concentration: 1.00 mg/liter) and 96.1 % for arsenic (V) (initial concentration: 1.25 mg/L). Importantly, nZVI/BC demonstrated successful treatment of real arsenic contaminated groundwater with a removal efficiency of 93 %. The arsenic removal mechanism involved electrostatic interactions, H-bonding, and electron transfer reactions, driving the reduction of arsenic (III) to less toxic forms. Importantly, nZVI/BC demonstrated successful treatment of real arsenic-contaminated groundwater. Characterization techniques, including SEM-EDX, TEM, and FTIR, confirmed porous surfaces with functional moieties involved in the adsorption process. nZVI/BC, analyzed by EDX before adsorption, exhibited Fe (18.0 %), Ca (11.4 %), O (63.9 %), and K (6.6 %), affirming ZVI layer formation on biochar. Post-adsorption, EDX revealed significant arsenic peaks, confirming successful arsenic uptake, with 6.1 % arsenic (III) and 4.2 % arsenic (V), indicating additional adsorption capacity. The adsorbent’s FTIR examination showed variations in peaks (from 3156 to 3175 cm⁻^1^, for example) that were suggestive of the participation of N–H amino groups in arsenic (III) and arsenic (V) adsorption. The binding sites of biochar, like C–H and C=O, showed a shift, indicating a major role of these functional groups in binding arsenic species. These findings underscore the potential of nZVI/BC as a viable option for large-scale arsenic remediation due to its economic feasibility and impressive removal performance, offering a promising alternative to current commercial adsorbents.

Another study presents a low-cost solid waste-based adsorbent derived from ***Cassia fistula*** pods for efficient arsenic elimination. The bioadsorbent demonstrated the highest achievable removal efficiency for arsenic approximately 91 %, highlighting its effectiveness. The arsenic adsorption onto *Cassia fistula* pods bioadsorbent exhibited a rapid initial sorption rate investigated via the pseudo-1st-order kinetic, while the Langmuir-model fitting indicated monolayer sorption behavior with a highest capacity of 1.13 mg/g [114]. The exceptional effectiveness of *Cassia fistula* biomass for arsenic removal establishes its prominence among biomass-derived adsorbents. Chunhui et al. study explores the utilization of ***yak dung***-derived biochar targeting the removal of arsenic from geothermal waters, a pressing environmental concern in the Tibetan region [115]. Biochar (BC_1_, BC_2_, and BC_3_) were produced via pyrolysis under varying settings, with BC_3_ exhibiting the highest efficiency, removing 20 % arsenic (V). Further modification of BC_3_ with FeCl_2_ (Fe/BC_3_) significantly enhanced its performance, achieving 99.45 % arsenic (V) elimination with biochar dosage of 10 g/L, pH 5–6, and 25 °C. The quasi-second-order kinetic aptly investigated the arsenic uptake capacity of the adsorbent, emphasizing a rapid initial sorption rate. The Langmuir model suggested monolayer sorption behavior, with the highest monolayer capacity of sorption of 2.926 mg/g (As (V)). Characterization through XRD, FTIR, SEM, and EDS revealed the structural and compositional changes in Fe/BC_3_, indicating the incorporation of Fe into the biochar structure. This study underscores the efficacy of yak dung-derived biochar, particularly Fe/BC_3_, as promising and sustainable adsorbents for arsenic removal in geothermal water, offering potential solutions for water remediation in the region.

**Fig. 9:**
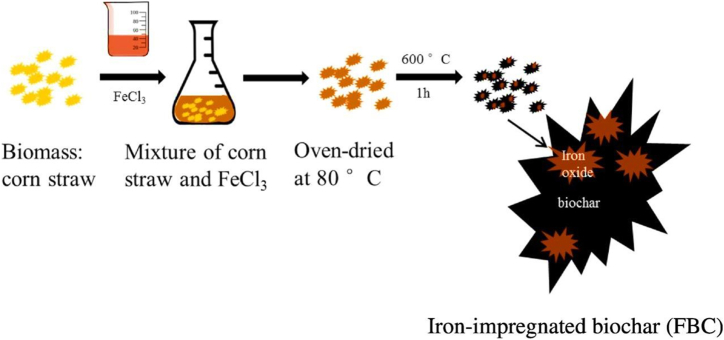
A Fabrication strategy for FBC composite. Reprinted with permission from Ref. [116].

**Fig. 10:**
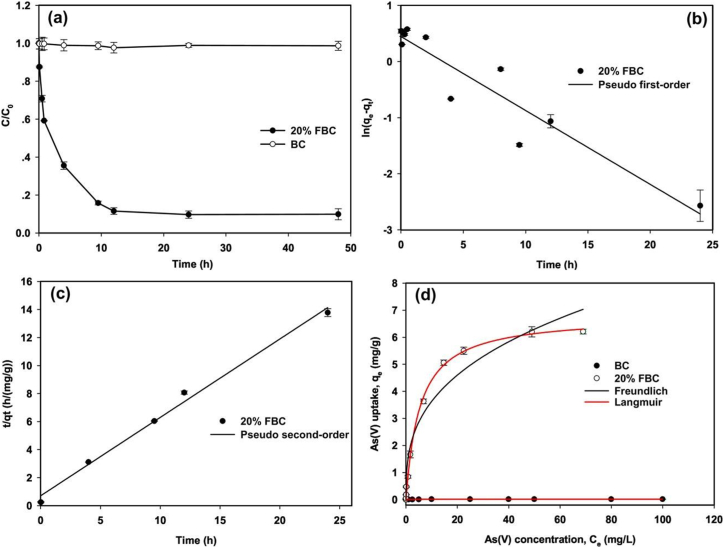
(a) Evaluation of As(V) sorption kinetics using kinetic models: (b) pseudo-1st -order and (c) pseudo-2nd-order approaches. Investigation of As(V) sorption by 20 % FBC. (d) Comparison of As(V) sorption isotherms for BC and 20 % FBC. Reprinted with permission from Ref. [116].

**Corn straw**-Fe-impregnated biochar (FBC) [116], synthesized with varying Fe concentrations (Fig. 9), displayed enhanced arsenate (As (V)) adsorption, reaching a maximum efficiency of 6.80 mg/g reflecting a substantial 400-times enhancement compared to unmodified biochar (BC). The adsorption process adhered to the pseudo-2nd-order kinetic, exhibiting a rate constant of 0.053 g/mg.h (Fig. 10a-c). Both Langmuir and Freundlich models aptly described the equilibrium sorption, manifesting peak capacities or sorption of 8.92 and 7.77 mg/g, respectively (Fig. 10d). Structural analyses confirmed the integration of iron oxide particles within the biochar matrix, augmenting surface area (297.13 m^2^/g) and porosity. The 20 % Fe concentration was optimal, rendering an iron content of 6.05 %. The FBC exhibited pH-dependent adsorption, with maximal efficiency (86.12 %) at pH 6.0, and PZC pH of 7.38. Notably, FBC demonstrated resistance to variations in ionic strength, while phosphate exerted a deleterious influence on arsenic (V) elimination. The regenerable nature of the FBC was evident, maintaining approximately 70 % removal efficiency over three successive cycles, solidifying its potential as a robust, environmentally friendly adsorbent for arsenic remediation.

***Waste orange peel***-derived calcined magnetic composites (CMOPC) possess large area of surface and size of void exhibit outstanding As(III) capacity at 10.3 mg/g [117]. Langmuir model and pseudo-2nd-order kinetics describe the equilibrium and kinetics, respectively. CMOPC displays excellent anti-interference ability, efficient regeneration (89 % after 4 cycles), and lowers arsenic(III) concentration to meet WHO standards in contaminated water. This study positions CMOPC as having economic viability and high removal efficiency addressing the global health hazard of arsenic contamination.

***Tectona and Lagerstroemia speciosa waste*** leaf-derived biochar (TB 800, LB 800) at 800 °C, with crystalline structures show effective arsenic removal (As(V): TB 800–94.6 %, LB 800–85.85 %) with significant capacities (As(III): TB 800–666.7 μg/g, LB 800–454.54 μg/g; As(V): TB 800–1250 μg/g, LB 800–714.28 μg/g) underscores the promising practical applications of these biomass [29].

The other study investigates the removal of arsenite (As(III)) and arsenate (As(V)) from aqueous solutions using magnetic biosorbents derived from waste Citrus limetta biomass. The biosorbents, designated PAC-500 and PPAC-500, were produced through a pyrolysis process at 500 °C, with adsorbent capacities of 714.28 μg/g and 526.31 μg/g (As(III)), and 2000 μg/g (As(V)) [118]. Characterization through BET, Zeta potential, FTIR, SEM, EDS, XRD, and PSA confirms magnetic properties and structural features. Regeneration study shows recyclability for up to four cycles. Groundwater treatment achieves over 88 % removal of As(V) and As(III). The isotherms follow Langmuir (PAC-500) and Temkin/Freundlich (PPAC-500) models, while kinetics adheres to pseudo-second order. The removal of arsenic (III) by PAC-500 occurred through an exothermic adsorption process, while the adsorption of arsenic (V) by PAC-500 and both arsenic (III) and arsenic (V) by PPAC-500 were endothermic. The negative ΔG° values for arsenic (V) with PAC-500 and for both arsenic (III) and arsenic (V) with PPAC-500 indicated that these sorption processes were spontaneous and thermodynamically favorable. The study contributes practical insights for the development of sustainable, magnetic biosorbents in arsenic removal applications.

The ability of six different biosorbents ***(shell of egg, seeds of java-plum, shell of water chestnut, corn-cobs, tea-waste, and peels of pomegranate)*** to eliminate arsenic(V) and arsenic (III) was assessed [119]. Notably, shell of egg and seeds of java plum exhibited robust elimination of arsenic (III) (78–87 % at 7 pH) within a 2-h time frame. The adsorption experiment followed a Langmuir model to represent the relationship between adsorbent and adsorbate at equilibrium, and a pseudo-2nd-order model to describe the rate of adsorption over time, implicating the role of surface functional groups in arsenic removal. Shell of egg and seeds of java plum demonstrated efficacy in multiple sorption/desorption cycles, presenting a promising, cost-effective solution for arsenic removal.

**Fig. 11:**
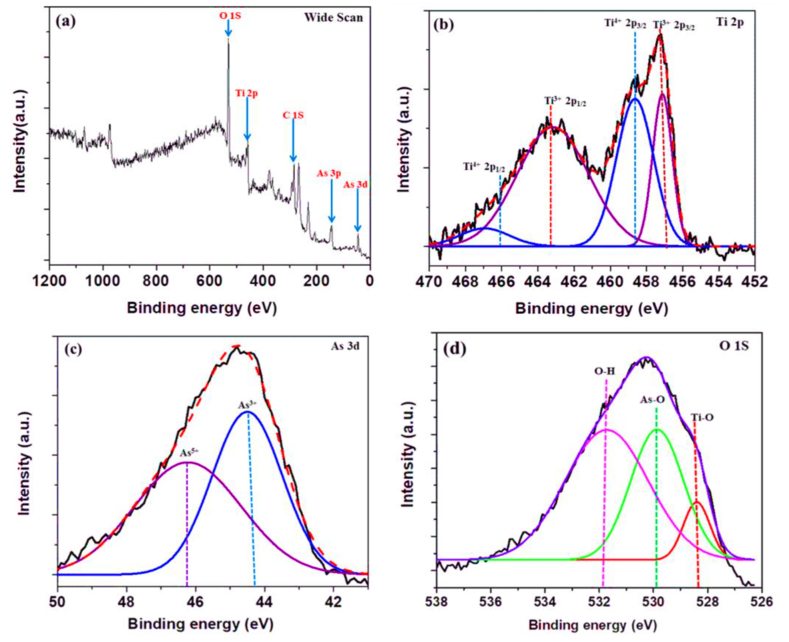
XPS spectra of PP@TiO_2_ Post As (III) sorption-XPS spectra of PP@TiO_2_: (a) Wide scan; (b) 2p Ti; (c) 3d As; (d) 1s O. Reprinted with the permission from Ref. [120].

Another study use ***pomegranate peel***, TiO_2_-impregnated peels of pomegranate (PP@TiO_2_) were prepared and characterized for arsenic(III) removal [120]. Effective at neutral pH, PP@TiO_2_ exhibited the highest capacity of 76.92 mg/g, following Langmuir isotherm and kinetics of pseudo-2nd-order. X-ray Photoelectron Spectroscopy (XPS) (Fig. 11a–d) showed that arsenic was found in both arsenic (III) and partially as arsenic (V) forms after adsorption. The PP@TiO_2_ material is effective even with other anions present and can be regenerated for reuse. Overall, PP@TiO_2_ emerges as a safe, economically viable adsorbent for efficient arsenic (III) removal from H_2_O.

***Polyalthia longifolia leaf powder (PLP)*** demonstrated efficient adsorption of arsenic(III) and arsenic(V) from a water environment, exhibiting maximum capacities of 1.76 mg/g and 1.87 mg/g, respectively, following Langmuir model [121]. Thermodynamic evaluation revealed a spontaneous and exothermic process, suggesting the potential practical application of PLP in arsenic removal.

Biochar derived from ***corn cob and coffee husk***, impregnated with ZnO, demonstrated a significantly increased capacity for arsenic(V) adsorption compared to the non-modified biochar [122]. The corncob-based ZnO-impregnated biochar (CC–ZnO) displayed a high maximum equilibrium capacity of adsorption for arsenic(V), reaching 25.9 mg/g.

Various other biomass-derived materials, including ***Java plum and amaltash seeds, hazelnut shells, rice husks, bamboo, cotton stalks, and crop straw***, have exhibited promising potential for arsenic remediation, as evidenced by a comprehensive analysis of pertinent literature spanning the years 2018–2024 (Table 1). In conclusion, the diverse range of biomass-derived materials explored in the presented studies demonstrates promising candidates for efficient and sustainable adsorbents for arsenic. From activated biochar to magnetic biosorbents and TiO_2_-impregnated pomegranate peels, these innovative approaches showcase promising results in terms of adsorption capacity, recyclability, and practical applicability. The comparative analysis underscores the versatility of these eco-friendly solutions, emphasizing their collective contribution to addressing the global challenge of arsenic contamination in aqueous environments.

**Table 1:** Biomass-derived adsorbents for arsenic remediation.

Adsorbent	Biomass material	Adsorption Isotherm study	Adsorption kinetics study	Maximum Capacity of adsorption (qe) mg/g or % removal for arsenic	Thermodynamics study	Year	Reference
Biochar	*Cassia fistula* (golden shower)	Freundlich isotherm	Pseudo 2nd order	0.78 or 78.1 % for arsenic (III) and 0.42 or 84.8 % for arsenic(V)	ΔG = -ve, ΔH = -ve ΔS = -ve, Exothermic and spontaneous	2018	[113]
Biochar	Yak dung	Freundlich isotherm (biochar) and Langmuir isotherm (Fe modified biochar)	Pseudo 2nd order	for arsenic(V) 99.45 % and 1.0497 (Unmodified biochar) 2.926 (Fe modified biochar)	–	2018	[115]
Biochar	Corn straw	Freundlich isotherm and Langmuir isotherm	Pseudo 2nd order	for arsenic(V) 6.80 (biochar modified with FeCl_3_) and 0.017 (unmodified biochar)	–	2018	[116]
Calcined magnetic orange peel composites	Orange peel	Langmuir isotherm	Pseudo 2nd order	10.3 for arsenic (III)	–	2018	[117]
Biochar	Waste leaves litter of *Tectona* (TB 800) and *Lagerstroemia speciosa* (LB 800)	Langmuir model for arsenic(V) (TB800 and LB800) and for arsenic (III) Freundlich model (LB 800) and Temkin model (TB 800)	Pseudo 2nd order	for arsenic(V) 1.25 (TB 800) and 0.71428 (LB 800) and for arsenic (III)0.6667 (TB 800) and 0.45454 (LB 800)	for arsenic(V) Exothermic and for arsenic(III) Endothermic	2019	[29]
Biosorbent PAC-500 and PPAC-500	*Citrus limetta* (peel and pulp)	Langmuir model for arsenic(V) and arsenic (III) (PAC-500) and Freundlich model for arsenic(V) and Temkin model for arsenic(III) (PPAC-500)	Pseudo 2nd order	for arsenic (III) 0.71428 (PAC-500), 0.52613 (PPAC-500) and for arsenic(V) 2 (PAC-500), and PPAC-500)	Exothermic for arsenic (III) (PAC-500) and Endothermic for arsenic (V) (PAC-500, PPAC-500) and for arsenic (III) (PPAC-500) and spontaneous	2019	[118]
Magnetic pine cone biomass	Pine cone	Langmuir model for arsenic(III)	Pseudo 2nd order	14.83 (pine cone biomass) and 18.02 (magnetic pine cone biomass)	–	2019	[77]
Biosorbents	Corn cob, water chestnut shell, java plum seed, tea waste, egg shell and pomegranate peel	Langmuir model	Pseudo 2nd order	for arsenic (III) 87 % (by egg shell) and 78 % (by java plum seed) for arsenic(V) 71 % (by egg shell) and 67 % (by java plum seed)	–	2019	[119]
Agro-Waste Derived Biomass Impregnated with TiO_2_	pomegranate peels	Langmuir isotherm	Pseudo 2nd order	for arsenic(III) 76.92	–	2020	[120]
Cellulosic biosorbent	*Polyalthia longifolia* Leaf powder	Langmuir isotherm	Pseudo 2nd order	1.76 for arsenic(III) and 1.87 for arsenic(V)	ΔG = -ve, ΔH = -ve ΔS = -ve, Exothermic and spontaneous	2020	[121]
Agrowaste derived biochars impregnated with ZnO	Coffee husk and Corncob	Langmuir model and Redlich-Peterson model	Elovich model	for arsenic(V) 25.9 (by corncob derived ZnO impregnated biochar)	–	2020	[122]
Algal biomass	*Turbinaria vulgaris*	Langmuir model and Dubinin–Radushkevich isotherm	Pseudo 2nd order	26.54 Or 92.12 %	–	2020	[123]
Biochar based iron nanocomposite	*Cassia fistula*	Langmuir isotherm model	Pseudo 2nd order	for arsenic (III) 1.04 or 99.1 % and for arsenic(V) 1.40 or 96.1 %	ΔG = -ve, ΔH = -ve ΔS = -ve, Spontaneous and exothermic	2020	[99]
Dried mixed bacterial biomass	*Bacillus thuringiensis Strain* WS3*, Pseudomonas stutzeri strain* WS9 and *Micrococcus yunnanensis strain* WS11	Langmuir isotherm model	Pseudo 2nd order	for arsenic (III) 11.92 or 95 % and for arsenic (V) 14.66 or 98 %	ΔG = -ve ΔH = +ve ΔS = +ve, Endothermic and spontaneous	2020	[126]
Java plum and amaltash seed biomass based bio-adsorbents	Java plum and amaltash seed	Freundlich and Tempkin models	Pseudo 1st order model or Elovich model.	1.45 or ∼93 % (by Java plum) and 1.42 or ∼91 % (by amaltash)	–	2021	[132]
Magnetic microporous biochar	Biomass hazelnut shell	Langmuir and Freundlich model	Pseudo 2nd order	(Organoarsenic compound p-arsanilic acid) 218.2 or 87.3 %	–	2021	[133]
Algal biomass	Chemically Modified *Chlorella vulgaris* and *Spirulina platensis*	Freundlich Model	Pseudo 2nd order	for arsenic(V) 20.9 (by *Chlorella vulgaris* modified with NaCl) and 24.8 (*Spirulina platensis* modified with ZnCl_2_)	–	2021	[124]
Phosphorus (P) modified biochar	Dietic herb *Taraxacum mongolicum* Hand-Mazz	Langmuir isotherm	Pseudo 2nd order	for arsenic (III) 30.76	–	2021	[134]
Magnetic biochar	Fresh bamboo	Langmuir isotherm	Pseudo 2nd order	for arsenic (III) 129.24 and for arsenic (V) 127.15	–	2022	[135]
Bioadsorbent	*Cassia fistula* pod	Freundlich model	Pseudo 1st order model	for arsenic (III) 1.13	–	2022	[114]
Magnetic biosorbents	MPAC-500 and MPAC-600 (magnetic-activated carbons synthesized from the peel of *Pisum sativum* (pea) pyrolyzed at 500 °C and 600 °C temperatures, respectively)	Langmuir isotherm (for arsenic (III) by MPAC-500 and MPAC-600 and As (V) by MPAC-500) and Freundlich isotherm (arsenic(V) by MPAC-600)	Pseudo 2nd order	for arsenic (III) 0.7297 (by MPAC-500) and 1.3335 (by MPAC-600) and for As (V) 0.4930 (by MPAC-500) and 0.9451 (by MPAC-600)	arsenic (III) by MPAC-500: endothermic And arsenic (III) by MPAC-600: exothermic and arsenic (V) by MPAC-500 and MPAC-600: endothermic	2022	[4]
Rice husk ash adsorbent modified by iron oxide	Rice husk	Langmuir isotherm	Pseudo 1st order model	25.06 Or 98 %	–	2022	[136]
Dried Bacterial Biomass	*Bacillus thuringiensis* strain WS3	Nonlinear Langmuir model	Nonlinear Pseudo 2nd order	27.28	–	2022	[137]
Biochar	*Mangifera indica* (M), *Artocarpus heterophyllus* (JF), and *Schizizium commune* (JP).	Freundlich model	Pseudo 2nd order	(M) 0.365 or 94 %, (JF) 29.25 or 93 %, and (JP) 0.360 or 92 %	–	2022	[138]
Metal oxide/chicken egg shell biomass	Waste biomass of *Gallus gallus* domesticus (Chicken), egg shell	Langmuir isotherm	Pseudo 2nd order	for arsenic (III) 40.0	–	2022	[139]
Biochar coated with iron	Pomelo peel	Redlich–Peterson model	Pseudo 2nd order	for arsenic (III) 11.77 and for arsenic (V) 15.28	–	2022	[140]
Cotton stalk biochar	Cotton stalk	Langmuir isotherm	–	for arsenic (III) 0.10278	–	2022	[141]
Calcium/*Moringa oleifera* wood biochar	*Moringa oleifera* wood	Langmuir isotherm and D-R isotherm	Pseudo 2nd order	for arsenic (III) 33.44 and for arsenic (V) 37.22	ΔG = -ve ΔH = -ve ΔS = -ve Spontaneous and exothermic	2022	[142]
biosorbent Dried microalga	Microalga *Chlamydomonas* sp.	Langmuir isotherm	Initial arsenic concentration 25 mg/L: 2nd order, for 50,180 mg/L: 1st order, for 100, 140, 200 mg/L: Elovich model	53.8 and 95.2 %	ΔG = -ve, ΔH = +ve ΔS = +ve, Endothermic and spontaneous	2022	[125]
Rice husk ash adsorbent modifed by iron oxide	Rice husk	Langmuir and Freundlich isotherm	Pseudo 2nd order	25.06	–	2023	[136]
Biochar	Crop straw (wheat), kitchen waste, leaf litter (sal), invasive plant (*Lantana*), fruit peel (orange), and dried fruit waste (walnut),	Freundlich isotherm	Pseudo 2nd order and Weber–Morris intra-particle diffusion model	for arsenic (III) 1.8 (Crop straw (wheat)), 2.5 (kitchen waste), 2.7 (leaf litter (sal)), 3.9 (invasive plant (*Lantana*)), 6.5 (fruit peel (orange)), and 2.8 (dried fruit waste (walnut))	ΔG = -ve, ΔH = -ve ΔS = -ve, Spontaneous and exothermic	2023	[143]
Chemically modified sugarcane bagasse	Sugarcane bagasse	–	–	27.4 ± 0.8	–	2023	[144]
Iron oxide impregnated *Pongamia pinnata*	*Pongamia pinnata*	Langmuir isotherm	–	for arsenic (III) 13.87 and for arsenic(V) 10.29	–	2023	[130]
Waste bamboo framework/α-FeOOH nanoneedles	Bamboo-Waste	Langmuir isotherm	Pseudo 2nd order	for arsenic(III) 35.69 and for arsenic(V) 20.35	–	2023	[145]
Microalgae	*Chlorella vulgaris*	Langmuir and Dubinin–Radushkevich isotherms	Pseudo-first order model	for arsenic (III) 55 and 100 %	ΔG = -ve, ΔH = +ve ΔS = +ve, Endothermic and spontaneous	2023	[146]
Peanut shell biochar (PSB) and modified peanut shell biochar (MPSB)	Peanut shell	Freundlich isotherm	Pseudo-second-order	for arsenic (III) 1.302 (by PSB),1.455 (by MPSB) and for arsenic (V) 1.031 (by PSB), 1.455 (by MPSB)	ΔG = -ve, ΔH = +ve ΔS = +ve, Endothermic and spontaneous	2023	[147]
Tea waste magnetic biosorbent (biochar)	Tea waste	Langmuir	Pseudo 2nd order	714 (arsenic (III))	ΔG = -ve (except 298 K) ΔH = +ve, ΔS = +ve Endothermic and spontaneous	2024	[7]

### 3.4 Algal, fungal, and bacterial biomass-derived adsorbents

Algae, with abundant polysaccharides and proteins, excel in arsenic adsorption, especially with enhanced capacity through pretreatments. Fungal biomasses, rich in sugars and functional groups, show promise for arsenic removal in wastewater. With its small size and adaptability, bacterial biomass offers effective arsenic adsorption in bioremediation applications using various mechanisms. Overall, algae, fungi, and bacterial biomass present diverse and promising avenues for arsenic adsorption in environmental remediation.

***Turbinaria vulgaris*** biomass demonstrated a removal efficiency of 92.12 % for arsenic ions, optimized at pH 4.41, biomass concentration of 0.3 g/L, starting arsenic amount of 21.33 mg/L, and temperature of 298.32 K. FTIR and SEM analyses confirmed the biomass’s chemical properties and morphology, highlighting metal ion presence post-biosorption [123]. The biosorbent exhibited a maximum theoretical arsenic biosorption potential of 26.54 mg/g, emphasizing its cost-effectiveness and eco-friendly nature. Kinetic studies revealed a chemisorption mechanism with surface ion exchange, establishing *T. vulgaris* as a promising sorbent for efficient metal arsenic removal.

Another study investigated arsenic(V) biosorption by ***Chlorella vulgaris*** and ***Spirulina platensis*** [124], assessing unmodified and chemically modified biomasses. Modification with NaCl and ZnCl_2_ resulted in the greatest improvement in the biosorbent’s capacity. Maximum capacities for arsenic (V) were 20.9 mg/g (*C. vulgaris modified by NaCl*) and 24.8 mg/g for ZnCl_2_-modified *S. platensis*. Pseudo-2nd-order kinetics explained the sorption process, and SEM and FTIR analyses revealed surface changes and functional groups contributing to improved biosorption. This investigation demonstrates the prospects of functionally treated algae biomasses for efficient arsenic removal from water. ***Chlamydomonas* sp.** exhibited effective arsenic (III) biosorption from aqueous solutions under optimized conditions (pH (4), temperature (25 °C), contact time (60 min), and biomass dosage (0.6 g/L)) [125]. The maximum removal percentage was 95.2 %, with a biosorption capacity of 53.8 mg/g. Characterization studies confirmed the biosorption process. Kinetics, isotherm equilibrium, and thermodynamics analyses supported *Chlamydomonas* sp. as a potent, reusable, cheap, and economically viable biosorbent for arsenic (III) remediation.

This section focuses on fungal biomass-derived adsorbents, we explore the arsenic(V) biosorption capabilities of chemically modified mycelia fungi biomasses [32], including ***Mucor sp-1, and 2, Cladosporium* sp.*, Paecilomyces* sp.*, Aspergillus fumigatus I–II and Aspergillus flavus III, IV, V***, coated with iron oxide. Noteworthy is the remarkable arsenic removal efficiency exhibited by *Aspergillus flavus IV, III, and V, Paecilomyces* sp.*, and Aspergillus fumigatus I,* attaining removal percentages of 97.1, 92.3 %, 90.3 %, 89.0 %, and 83.4 %, respectively, as determined by atomic absorption spectroscopy (AAS) at pH 6.0 and 30 °C after a 24-h incubation period with 1 g/100 mL of biomass based on fungal. The Langmuir isotherm and pseudo-second-order kinetic models were identified as the most suitable models of these fungal variants as promising remediation agents for metal-contaminated environments.

Three arsenic-resistant bacterial strains: Gold mine tailing dam sludge served as the source for the isolation of three bacterial strains: ***Bacillus thuringiensis WS3***, ***Pseudomonas stutzeri WS9***, and ***Micrococcus yunnanensis WS11*** [126]. Their mixed dried biomass achieved significant removal of both arsenic(III) and arsenic(V), with maximum efficiencies reaching 95 % and 98 %, respectively, under operating parameters: the experiment used an 8-h reaction time for arsenic(III) and a 6-h reaction time for arsenic(V). The temperature was set at 37 °C, the solution had a neutral pH of 7, and the amount of biosorbent used was 0.60 mg/mL. The Langmuir isotherm and pseudo-2nd-order kinetic models were identified as the most suitable models, with the highest capacities of 11.92 mg/g: arsenic(III) and 14.66 mg/g: arsenic(V). FESEM–EDX and FTIR investigation confirmed morphological changes and highlighted functional groups involved in arsenic adsorption. Artificial neural network modeling demonstrated high accuracy in predicting arsenic removal, endorsing the recommendation of mixed dried biomasses for arsenic bio-treatment.

### 3.5 Iron-modified biomass-derived adsorbent

Iron-based adsorbents have garnered attention for their effectiveness in arsenic removal, environmental sustainability, and plentiful availability. This review also focuses on innovative iron-based adsorbents with high arsenic adsorption capacities and outlines the mechanisms involved. It includes a summary of key literature on arsenic remediation through adsorption. The study explored the use of iron-modified biochar (FMBC) from rice straws for removing arsenic (V) from water [127]. The FMBC achieved a high adsorption capacity of 28.49 mg/g at pH 5.0, fitting well with the Langmuir isotherm and pseudo-second-order kinetic model. The mechanisms for arsenic (V) removal by FMBC involve complexation with iron, as illustrated by the following equations:

$$\text{FeOH}+H_{3}\text{As}O_{4}\to \text{Fe}H_{2}\text{As}O_{4}+H_{2}O$$

$$\text{FeOH}+H_{2}\text{As}O_{4}^{-}\to \text{Fe}H_{2}\text{As}O_{4}^{-}+H_{2}O$$

$$\text{FeOH}+\text{HAs}O_{4}^{2-}\to \text{FeAs}O_{4}^{2-}+H_{2}O$$

The results indicate FMBC’s potential as an efficient and cost-effective alternative to commercial adsorbents for arsenic removal in groundwater. In a recent study sugarcane straw-based biochar modified with FeCl_3_, shows good adsorptive performance for arsenic from water [128]. Another study presents a new method for simultaneously removing ROX (4-hydroxy-3-nitrobenzene arsonic acid) and arsenic (III/V) from synthetic aqueous solutions using FeCl_3_-modified sorghum straw biochar (MSSB) [129], showing excellent adsorption capacities for ROX, arsenic (III), and arsenic (V). The adsorption followed the pseudo-second-order equation and Langmuir isotherm. MSSB prove to be an effective, low-cost adsorbent for arsenic removal with significant potential for environmental restoration. The study developed a cost-effective magnetic bio-adsorbent from *Pongamia pinnata* hulls, a biodiesel byproduct, for arsenic removal from water. The biochar was magnetized with Fe_3_O_4_ nanoparticles, facilitating easy separation after adsorption [130]. Characterization techniques such as SEM, EDS, TEM, XRD, XPS, TGA, and FTIR confirmed its properties. Optimal adsorption conditions were determined to be 200 mg/L dosage, pH 7.0, 0.5 mg/L initial arsenic concentration, and 45 min contact time. The Langmuir isotherm model fit the data well, with maximum capacities of 13.87 mg/g for arsenic(III) and 10.29 mg/g for arsenic(V). This modification offers an effective alternative for arsenic removal in various environmental settings. One study evaluated the adsorption of dimethylated arsenicals on rice husk biochar (BC) and iron-modified biochar (FeBC) through isothermal adsorption experiments and X-ray absorption spectroscopy [131]. The Langmuir isotherm indicated maximum adsorption capacities of 1.28 mg/g for BC and 6.32 mg/g for FeBC for inorganic arsenate. Dimethylated arsenicals did not adsorb to BC, but FeBC showed qm values of 7.08 mg/g for DMA(V), 0.43 mg/g for DMMTA(V), and 0.28 mg/g for DMDTA(V). This effectiveness is attributed to the iron oxide (two-line ferrihydrite) on FeBC. X-ray absorption near edge structure spectra revealed that all dimethylated arsenicals adsorbed as DMA(V). Consequently, FeBC can immobilize highly toxic arsenicals like DMMTA(V) and DMDTA(V), converting them into the less toxic DMA(V).

In conclusion, algae, fungi, and bacterial biomass offer diverse and promising avenues for arsenic adsorption in environmental remediation, with each demonstrating unique capabilities and mechanisms. *Turbinaria vulgaris* and *Chlamydomonas* sp. algae, chemically modified *Chlorella vulgaris* and *Spirulina platensis*, and iron oxide-coated fungal biomasses exhibit high arsenic removal efficiencies. Additionally, arsenic-resistant strains of bacteria, including, *Micrococcus yunnanensis* WS11, *Pseudomonas stutzeri* WS9, and *Bacillus thuringiensis* WS3 showcase efficient deletion of both arsenic(III) and (V) ions. Iron-modified biomass-derived adsorbents, such as iron-modified biochar (FMBC) from rice straw, FeCl_3_-modified sorghum straw biochar (MSSB), and Fe_3_O_4_-magnetized *Pongamia pinnata* hulls, demonstrate high arsenic adsorption capacities and cost-effectiveness, following Langmuir isotherm and pseudo-second-order kinetics. These adsorbents effectively remove various arsenic species from water, offering sustainable alternatives for environmental restoration. FeBC also successfully adsorbs dimethylated arsenicals, converting them to less toxic forms. These findings highlight the potential of these biomasses as effective, reusable, and environmentally friendly biosorbents for arsenic remediation. Table 1 outlines a comprehensive array of biomass-derived adsorbents utilized for arsenic remediation spanning the years 2018–2024.

## 4 Adsorption mechanism of arsenic on biomass-based adsorbent

The adsorption mechanism of arsenic on biomass-based adsorbents involves both physisorption and chemisorption processes. Biomass-derived adsorbents are rich in different functional groups such as –CH_3_, –OH, NH_2_, –CH_2_-, O=C, O=C–O, -Fe-OH, and C=C, these facilitate physisorption through interactions including eelectrostatic attractions, diffusion, van der Waals forces, and hydrogen bonding. Chemisorption, alternatively, encompasses electronic and valence forces, chemical bonding, formation of complexes, chelation, proton exchange, oxidation-reduction reactions, and bonding through shared electrons.

Various biomass-based adsorbents, including iron-modified biomass, nZVI/BC [99], corn straw-derived adsorbent [116], CMOPC [117], TB 800, LB 800 [29], PAC-500, PPAC-500 [118], and modified peanut biochar [147], exhibit distinct arsenic adsorption mechanisms. For instance, nZVI/BC demonstrates enhanced adsorption due to the stabilizing matrix support of biochar, allowing effective electrostatic interactions and surface complexation with arsenic oxyanions. The sorption mechanism of corn straw-derived adsorbent involves chemical sorption, electrostatic attraction, and precipitation reactions forming iron arsenate. CMOPC adsorption combines Outer-sphere interactions, oxidation-reduction reactions, inner-sphere surface interactions, and complexation of surface. Arsenic adsorption on TB800 and LB800 relies on electrostatic interaction and complexation with surface functional groups, exhibiting pH-dependent behavior.

PPAC500 and PAC500 biosorbents present homogeneous-heterogeneous biosorption, respectively, influenced by diverse surface functional groups. Modified peanut biochar, with its multiple interactions such as hydrogen bonding, hydrophobic interactions, electron donation, electrostatic attraction, ion exchange, and co-precipitation, proves effective in arsenic removal. Green algae *C. vulgaris* [146] can adsorb As ions depending on pH, a process found to be endothermic and spontaneous according to thermodynamic studies. The biosorption behavior fits well with the Langmuir model. Analysis using FTIR shows that the algal surface contains carboxylate, hydroxyl, amide, and amino functional groups.

**Fig. 12:**
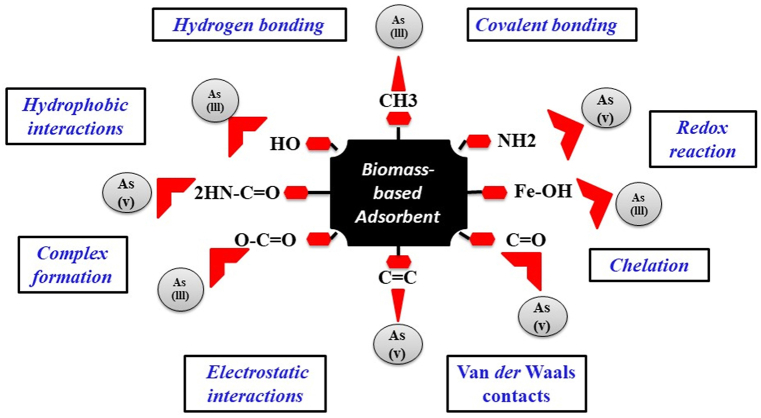
Adsorption mechanism of arsenic on biomass-based adsorbent.

In summary, the comprehensive understanding of diverse adsorption mechanisms on biomass-derived adsorbents highlights the intricate interplay of chemical and physical modification, providing valuable insights into development of efficient as well as sustainable arsenic removal materials and technologies. Further research is encouraged, especially in integrated spectroscopic techniques and life cycle assessments, to advance the field and address environmental concerns associated with arsenic remediation. Fig. 12 given below show the adsorption mechanism of arsenic on biomass based adsorbent.

## 5 Conclusion and future scopes

The diminution of arsenic-ions contamination in water is critical due to global health and environmental risks. Traditional industrial-level arsenic removal solutions are frequently challenging and expensive. However, the utilization of biomass-derived adsorbents has emerged as a viable, economical, and eco-conscious solution. This conclusion summarizes the efficacy of these materials, modified through processes such as alkali/acid treatment, pyrolysis, and composite formation with metal oxides, resulting in higher adsorption capabilities than pristine biomass. Recent advances in biomass-derived adsorbents can originate from diverse sources such as, including plant, animal, and farming waste, as well as algal, fungal, bacterial sources. The pH-dependent nature of arsenic ion species in aqueous solutions highlights the need for regulating pH for efficient adsorption, which typically ranges from 2 to 7. Adsorption on biomass-derived materials is a physical and chemical process that is supported by isotherm investigations, kinetic analyses, and thermodynamic evaluations, which are often based on Langmuir or Freundlich isotherms. While significant strides have been made in utilizing sustainable biomass-derived adsorbents for arsenic removal, addressing remaining challenges is imperative for future advancements in this field.

• *Comprehending adsorption mechanisms:* Prioritize improving our comprehension of adsorption mechanisms occurring at the interface between sorbent and water is crucial. Investigate molecular interactions to improve the design and performance of biomass-derived adsorbents.
• *The transition from batch to continuous systems:* Investigate and build continuous flow systems for real-world applications, expanding beyond laboratory-scale batch studies. Assess the performance and scalability of biomass-based adsorbents in continuous water treatment operations.
• *Cost Analysis and Life Cycle Examination:* Conduct cost comparisons to determine the economic viability of biomass-derived adsorbents vs. existing methods. Conduct life cycle assessments to determine these adsorption methods' environmental impact and sustainability.
• *Pilot Studies under Real-World Circumstances:* Conduct thorough pilot experiments in various real-world scenarios to validate the efficacy of biomass-derived adsorbents. Address issues and optimize parameters for a variety of water sources, climates, and pollutant levels.
• *Integrated spectroscopic techniques:* Investigate and apply integrated spectroscopic techniques to gain a thorough understanding of adsorption mechanisms. Use modern analytical tools to investigate the surface characteristics and structural changes of biomass-derived adsorbents after arsenic adsorption.
• *Regeneration and disposal methods:* Create efficient and eco-friendly ways for regeneration of arsenic-laden biomass-derived adsorbents. Establish safe and sustainable sludge disposal techniques that consider potential environmental implications.
• *Innovative and cost-effective solutions:* Foster innovation to overcome cost obstacles, especially in resource-constrained environments. Encourage strategic cooperation across academics, industry, and government to help develop and implement cost-effective solutions.
• *Dynamic, fixed-bed column study*: Future research will delve into the dynamic, fixed-bed column study of arsenic adsorption [148]. This investigation will assess the adsorbent’s performance under various operational conditions, focusing on optimizing flow rates and bed heights for maximum efficiency. Additionally, the long-term stability and regeneration potential of the adsorbent will be evaluated to ensure sustainability and cost-effectiveness. The outcomes will provide valuable insights for scaling up and implementing this technology in real-world water treatment applications.
• *Economic study*: The economic study of biochar and biosorbents derived from biomass for arsenic remediation highlights their cost-effectiveness and sustainable production. Raw material costs are low due to the use of agricultural residues, forestry waste, and industrial by-products. Production involves pyrolysis and potential chemical treatments, with associated costs for energy, labor, and equipment. The market value of biochar and chemically activated biosorbents offers a significant revenue potential. Additional benefits include by-products like bio-oil and syngas, and carbon credits from biochar production. Overall, these factors suggest promising financial viability for large-scale water treatment applications. However, none of the existing studies provide a detailed economic analysis of their materials.

Future research should include an economic analysis to assess the cost-effectiveness of using biochar and biosorbents derived from biomass for arsenic remediation. This analysis would help determine if these materials are financially viable options for large-scale water treatment. Additionally, understanding the economic feasibility will guide decision-making for implementing sustainable and affordable arsenic removal solutions.

To summarize, the future of arsenic removal using biomass-derived adsorbents requires a comprehensive approach that balances scientific advances with practical issues. The emphasis should be on understanding mechanisms, migrating to continuous systems, conducting real-world pilot studies, undertaking extensive assessments, economic study and addressing regeneration and disposal issues. This will facilitate the path for sustainable and successful arsenic removal solutions that preserve both the environment and public health.

## Data availability

No data associated with this study have been deposited into a publicly available repository. Data included in article/referenced in article.

## CRediT authorship contribution statement

**Gaurav Sharma:** Writing – review & editing, Writing – original draft, Conceptualization. **Yaksha Verma:** Writing – original draft, Formal analysis, Data curation. **Chin Wei Lai:** Writing – review & editing. **Mu Naushad:** Writing – review & editing. **Jibran Iqbal:** Writing – review & editing. **Amit Kumar:** Writing – review & editing. **Pooja Dhiman:** Writing – review & editing.

## Declaration of competing interest

The authors declare the following financial interests/personal relationships which may be considered as potential competing interests: Chin Wei Lai reports financial support was provided by 10.13039/501100004386Universiti Malaya Research Excellence Grant (UMREG). Chin Wei Lai reports financial support was provided by SATU Joint Research Scheme. If there are other authors, they declare that they have no known competing financial interests or personal relationships that could have appeared to influence the work reported in this paper.
